# Introducing GATA3 as a prominent player in Crohn’s disease 

**Published:** 2020

**Authors:** Sina Rezaei-Tavirani, Nastaran Asri, MohammadAli Emamhadi, Somayeh Jahani-Sherafat, Ali Seyed Salehi, Zahra Gholamrezaei, Elena Lak

**Affiliations:** 1 *Proteomics Research Center, Faculty of Paramedical Sciences, Shahid Beheshti University of Medical Sciences, Tehran, Iran*; 2 *Gastroenterology and Liver Diseases Research Center, Research Institute for Gastroenterology and Liver Diseases, Shahid Beheshti University of Medical Sciences, Tehran, Iran *; 3 *Forensic Medicine Department, Shahid Beheshti University of Medical Sciences, Tehran, Iran*; 4 *Laser Application in Medical Sciences Research Center, Shahid Beheshti University of Medical Sciences, Tehran, Iran *; 5 *Basic and Molecular Epidemiology of Gastrointestinal Disorders Research Center, Research Institute for Gastroenterology and Liver Diseases, Shahid Beheshti University of Medical Sciences, Tehran, Iran*

**Keywords:** Crohn’s disease, Genes, Gene ontology, GATA3 transcription factor

## Abstract

**Aim:**

This study was aimed at gene assessment of Crohn's disease (CD) through protein-protein interaction (PPI) network analysis to find crucial genes.

**Background:**

CD is a major subtype of inflammatory bowel diseases (IBD), which affects gastrointestinal tract. PPI network analysis is a suitable tool to clarify a critical gene as a drug target or diagnostic biomarker for these types of diseases.

**Methods:**

Gene expression profile GSE126124 of 20 CD patients and 20 healthy controls was obtained from the Gene Expression Omnibus (GEO) database. RNA profile of peripheral blood mononuclear cells (PBMCs) and colon biopsy samples of the studied groups was investigated. Crucial genes were selected and analyzed via the PPI network by Cytoscape software. Gene ontology enrichment for the hubs, bottlenecks, and hub-bottlenecks was performed via CluGO plugin of Cytoscape software.

**Results:**

Eighty-one differentially expressed genes (DEGs) among 250 initial DEGs were highlighted as significant by FC>2 and p-value ≤ 0.05, and 69 significant DEGs were used for PPI network construction. The network was characterized by poor connections, so 20 top neighbors were added to form a scale-free network. The main connected component included 39 query DEGs and 20 added first neighbors. Three clusters of biological processes associated with crucial genes were identified and discussed.

**Conclusion:**

The results of this study indicated that GATA3 has a key role in CD pathogenesis and could be a possible drug target or diagnostic biomarker for Crohn’s disease.

## Introduction

 Inflammatory bowel diseases (IBD) are chronic relapsing disorders with the ability to affect the entire intestine accompanied by long‐term morbidity (1-3). The two main subgroups of IBD are Crohn's disease (CD) and ulcerative colitis (UC) (4, 5). CD affects gastrointestinal (GI) tract and causes abdominal pain, fever, and clinical signs of bowel obstruction (5-8). A combination of genetic and environmental risk factors associated with CD susceptibility and numerous studies have pointed to the role of different gene groups in the pathophysiology of this disease (9-12). Different genetic studies using molecular biology, high throughput methods, and bioinformatics approaches tried to discover the molecular mechanism and biochemical pathways involved in CD development (13, 14). Protein-protein interaction (PPI) network analysis is an approach to evaluate dysregulated genes (queried genes) in patients compared to healthy individuals through a scale-free network. The topological properties of the created scale-free network contained useful information to discriminate queried dysregulated genes (15, 16). Centrality measures such as “Degree centrality (K)” and “Betweenness centrality (BC)” are used in analyzing the role of network elements. Degree refers to the number of the first neighbor of a node (equal to the number of links between the studied nodes with its first neighbors) and BC is a function of the shortest path where the node is involved. Degree and BC are widely used to analyze the studied network (17). Based on the degree value and BC amount, three types of critical nodes can be determined: the hub, bottleneck, and hub-bottleneck nodes. The top nodes based on degree value and BC are known as hub and bottleneck, respectively. Hub-bottleneck nodes are regarded as the common hub and bottleneck elements of the network.

This study aimed to find a possible new biomarker for Crohn’s disease through analyzing and screening related genes. The crucial genes were enriched via the gene ontology method. 

## Methods

Gene expression profile GSE126124 of a total of 20 CD patients (10 males and 10 females) and 20 healthy controls (10 males and 10 females) were extracted from Gene Expression Omnibus (GEO) database. RNA profile of peripheral blood mononuclear cells (PBMCs) and colon biopsy samples from patients with CD vs. controls was investigated. The age of patients and controls were between 8-18 years old. Gene expression distributions were evaluated through boxplot analysis using GEO2R. The top 250 differentially expressed genes (DEGs) based on p-value amounts were selected for more investigation. Eighty-one DEGs that were characterized by Fold change (FC) > 2 and p-value ≤ 0.05 were considered as significant DEGs. The significant DEGs plus the 20 first neighbors were included in a PPI network by Cytoscape software v 3.7.2 and STRING. The constructed network was analyzed by Network analyzer (an application of Cytoscape) to identify crucial genes. Degree and Betweenness centrality (BC) were considered to find critical DEGs. The top 10% of the main connected component network based on degree value and BC were considered as hub and bottleneck nodes, respectively. The nodes that were both hub and bottleneck were determined as hub-bottlenecks. Gene ontology enrichment for the hubs, bottlenecks, and hub-bottlenecks was performed via the CluGO plugin of Cytoscape software. The significant biological terms based on p-value ≤ 0.05 were identified. The terms were created in three groups, which were connected to hubs, bottlenecks, and hub-bottleneck. 

## Results

**Figure 1:**
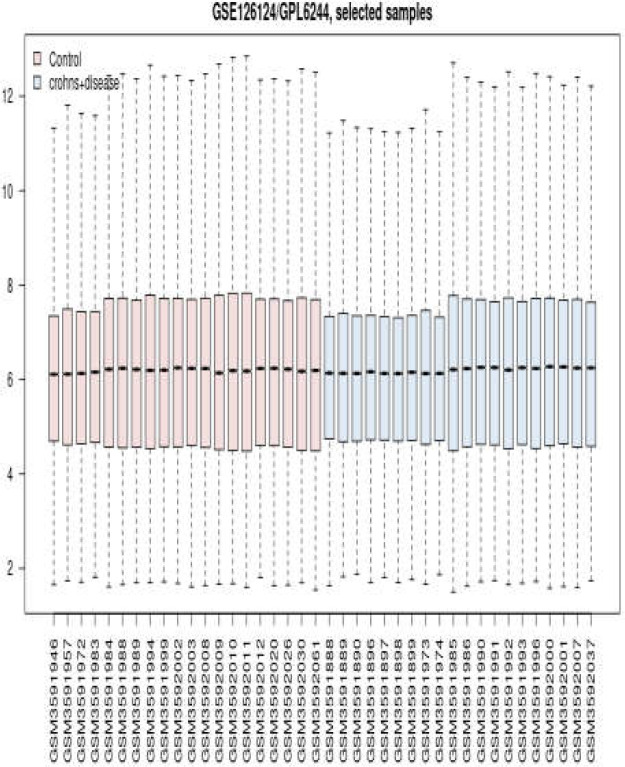
Distribution of gene expression changes in CD patients and controls. Blue-colored bars: CD patients | Pink-colored bars: Controls

Gene expression profiles of 20 CD patients and 20 controls were matched by boxplot analysis (Figure 1). 

**Figure 2:**
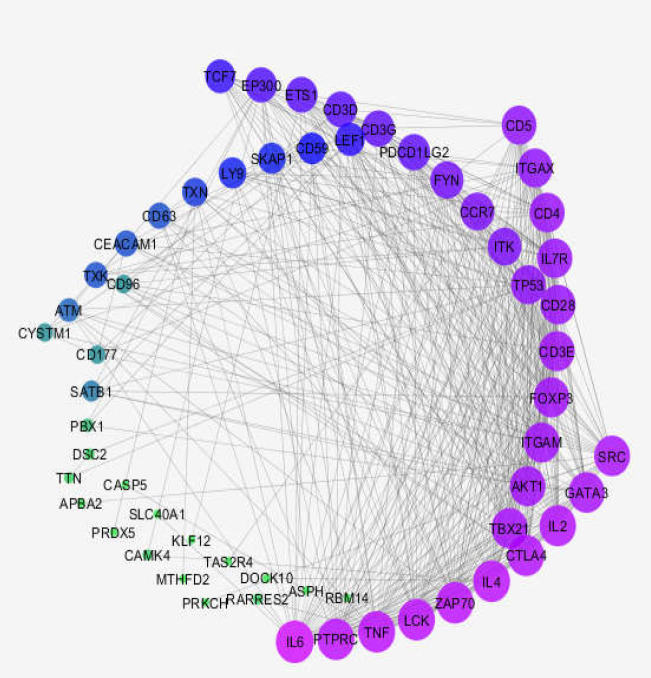
The main constructed PPI network. The 59 genes (39 queried DEGs and 20 first neighbors) were constructed as a PPI network. The network is a layout by the degree value of the nodes. The larger size of the node corresponds to a higher value of the degree. The color change from purple to green indicates an increment of degree value

Since the middle line of samples was matched, the studied profiles were comparable. The top 250 DEGs, based on p-value, were identified by the GEO2R analyzer. Among them, 81 DEGs were characterized as the significant DEGs by FC>2 and p-value ≤ 0.05. Sixty-nine (85%) of the 81 significant DEGs were recognized by the STRING database and were candidate to construct the PPI network. The created network was not considered as scale-free because of the weak interactions between the included DEGs. As most of the queried DEGs were isolated, 20 first neighbors were added to the queried DEGs to form a scale-free network (Figure 2). 

Thirty isolated query DEGs were excluded from further investigation, and therefore the constructed scale-free network included 59 linked DEGs, 31 of which were identified as connected components. Hence, the main connected component included 39 query DEGs and 20 added first neighbors, and the central nodes were selected among the 39 query DEGs for further evaluations. To investigate which nodes play a more important role in the main connected component constructed network, two central parameters (degree and BC) were considered. The top 10% (4 individual) of nodes based on degree value were introduced as the hub nodes. The bottleneck nodes were identified as the top 10% of nodes based on betweenness centrality. The hubs (GATA3, IL-7, CD28, CD5) and bottlenecks (TXN [Thioredoxin], ETS-1, GATA3, CCR7 [CC-chemokine receptor 7]) are presented in table 1 and 2, respectively.

**Table 1:** Top 10% of queried nodes based on the highest degree value presented as hub nodes

*R*	*Name*	*K*	*BC*
*1*	GATA3*	28	0.038
*2*	IL-7	26	0.007
*3*	CD28	26	0.006
*4*	CD5	25	0.005

* hub-bottleneck

**Table 2:** Top 10% of queried nodes based on the highest betweenness centrality presented as bottleneck nodes

*R*	*Name*	*K*	*BC*
*1*	TXN	9	0.069
*2*	ETS-1	19	0.042
*3*	GATA3*	28	0.038
*4*	CCR7	23	0.036

* hub-bottleneck

**Figure 3:**
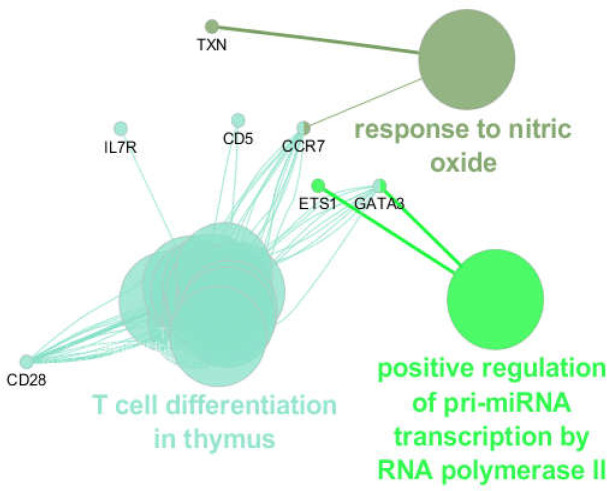
A total of 18 biological terms and pathways that are classified into 3 groups (response to nitric oxide, positive regulation of pri-miRNA transcription by RNA polymerase II, T cell differentiation in the thymus) in relationship with the hubs, bottlenecks, and hub-bottleneck

 These seven genes have been considered crucial genes, and since GATA3 is common in hubs and bottlenecks, it is termed hub-bottleneck. Three clusters of biological processes related to the seven crucial genes were determined (figure 3). For better understanding, the biological processes were screened to find the role of crucial genes (Table 3). 

## Discussion

The proteomic studies have provided valuable information about the molecular mechanism of disorders leading to the development of therapeutic and diagnostic methods (18). Results of proteomic or genomic studies contain a large amount of differentially expressed genes/ proteins (DEGs/ DEPs) between patients and healthy controls. As a bioinformatics approach, PPI network analysis is closely tied to proteomics and genomics investigations (19, 20). In this study, PPI network analysis is applied to screen the known proteins or genes related to Crohn’s disease. Among the 81 queried DEGs related to Crohn’s disease, 7 genes (IL-7, GATA3, CD28, CD5, TXN, ETS-1, and CCR7) were introduced as dysregulated genes. 

**Table 3:** The biological terms and pathways concerning hubs, bottlenecks, and hub-bottleneck genes including IL-7, GATA3, TXN, ETS-1, CCR7, CD28, and CD5

R	Goterm	gene name
1	response to nitric oxide	CCR7, TXN
2	positive regulation of pri-miRNA transcription by RNA polymerase II	ETS-1, GATA3
3	T cell differentiation in thymus	CCR7, CD28, GATA3, IL-7
4	inflammatory response to antigenic stimulus	CCR7, CD28, GATA3
5	interleukin-4 production	CD28, GATA3
6	acute inflammatory response to antigenic stimulus	CCR7, GATA3
7	lymphocyte costimulation	CCR7, CD28, CD5
8	regulation of inflammatory response to antigenic stimulus	CCR7, CD28
9	regulation of interleukin-4 production	CD28, GATA3
10	hypersensitivity	CCR7, GATA3
11	positive regulation of inflammatory response to antigenic stimulus	CCR7, CD28
12	positive regulation of interleukin-4 production	CD28, GATA3
13	T cell costimulation	CCR7, CD28, CD5
14	T cell selection	CCR7, CD28, GATA3
15	negative T cell selection	CCR7, CD28
16	thymic T cell selection	CCR7, CD28, GATA3
17	Selective expression of chemokine receptors during T-cell polarization	CCR7, CD28
18	negative thymic T cell selection	CCR7, CD28

Dysregulation of cytokines and growth factors play a major role in the malfunction of the immune system in inflammatory bowel disease (21). IL-7 signals are involved in the development of chronic intestinal inflammation and human intestinal diseases (22). As depicted in figure 3 and table 3, IL-7 is related to the T cell differentiation in the thymus, which is the dominant class of biological terms and contains 16 biological terms (about 89% of all known terms). According to the results of studies, the lymphocyte population is reduced in the peripheral blood of CD patients, which is due to a decrease in IL-7 levels (23). Kader et al. (24) reported significantly higher levels of IL-7 in CD patients in remission compared to patients with active form.

GATA3 is the only hub-bottleneck node that is characterized by degree value=28 and BC=0.038. Hub-bottleneck nodes are known as the key element of the PPI network. There are several studies on the role of the hub-bottleneck nodes in the incidence and development of diseases (16). Like IL-7, GATA3 is involved in T cell differentiation in the thymus. Moreover, GATA3 plus ETS-1 are the two dysregulated genes associated with positive regulation of pri-miRNA transcription by RNA polymerase II. GATA3 is a transcription factor with the ability to control Foxp3 (Forkhead box P3) expression and regulatory T (Treg) cells function. GATA3 is an essential factor for immune tolerance, and its removal is associated with autoimmune diseases (25). Li et al. (26) reported decreased Gata3+ Foxp3+ cell population in severe CD patients compared to controls.

Research has shown that TXN is involved in various biological pathways and processes (27). TXN activities include the protection of proteins against aggregation and oxidative stress, helping cells to encounter environmental stresses. They also have a direct role in apoptosis, inflammatory processes and cell growth (28-31). Nitric oxide pathway is a well-recognized biological term in Crohn’s disease (32). In our investigation, the role of TXN in response to nitric oxide is highlighted. Oxidative stress has an important role in IBD pathogenesis and reduced and oxidized thioredoxin have a key role in intestinal redox biology (33, 34). Tamaki et al. (35) demonstrated increased serum thioredoxin levels in IBD patients (both UC and CD) related to controls and its correlation with their disease activity.

As mentioned before, ETS-1 is associated with the positive regulation of pri-miRNA transcription by RNA polymerase II. The ETS-1 transcription factor can regulate the expression of pro-inflammatory cytokines like IFN-γ (Interferon-gamma) and TNF-α (Tumor Necrosis Factor Alpha) and is related to the disease activity of IBD patients (36). Li et al. (37) reported over-expression of ETS-1 in intestinal epithelial cells (IECs) of UC patients.

The chemokine receptor CCR7 expression controls the entry of naive CD4+ T cells into secondary lymphoid organs and evidence suggests a relationship between its expression and intestinal inflammation (38-40). Kawashima et al. (41) found a significant rise in CCR7 mRNA expression in CD patients compared to UC subjects and healthy controls.

CD28 is a differentiation antigen expressed on T cells and plays a role in Treg-mediated autoimmunity control (42, 43). Recent research has revealed association of CD28 downregulation with the activity index of Crohn’s disease. In fact, the larger number of memory T cells accompanied by downregulated CD28 expression is introduced as a sign of active Crohn’s disease (44).

CD5 is a scavenger-like receptor expressed on T and B-1a lymphocytes with the ability of TCR (T-cell receptor) and BCR (B-cell receptor) signaling negative regulation (45, 46). Using cytokines like IL-10, CD5 positive cells have a role in the prevention of autoimmunity (47). Voisinne et al. (46) found a smaller number of CD5+ B cells in CD patients in comparison to normal controls.

As it is discussed, intestinal inflammation, immune tolerance, intestinal redox biology balance, CD activity index, autoimmunity control, and consequently, the development of CD related presentations are the main processes associated with the introduced seven critical genes (IL-7, GATA3, CD28, CD5, TXN, ETS-1, and CCR7). Evaluation of these critical genes can lead to finding the key element as a drug target or diagnostic biomarker for Crohn’s disease. In this study, GATA3 was highlighted as the most important CD-related gene with a dramatic difference in expression appearing as a single hub-bottleneck of the network.

Through network analysis, we can introduce IL-7, GATA3, TXN, ETS-1, CCR7, CD28, and CD5 as Crohn’s disease-related critical genes and possible biomarker panel. GATA3 was highlighted as the key element among them, and it was concluded that GATA3 might be a possible drug target or diagnostic biomarker for the disease. Notably, these differentially expressed genes can be investigated in greater detail in further studies with larger number of CD patients.
